# Effect of Topical Tranexamic Acid on Bleeding and Quality of Surgical Field during Functional Endoscopic Sinus Surgery in Patients with Chronic Rhinosinusitis: A Triple Blind Randomized Clinical Trial

**DOI:** 10.1371/journal.pone.0104477

**Published:** 2014-08-18

**Authors:** Javaneh Jahanshahi, Farnaz Hashemian, Sara Pazira, Mohammad Hossein Bakhshaei, Farhad Farahani, Ruholah Abasi, Jalal Poorolajal

**Affiliations:** 1 Department of Ear-Nose-Throat Surgery, School of Medicine, Hamadan University of Medical Sciences, Hamadan, Iran; 2 Department of Anesthesiology, School of Medicine, Hamadan University of Medical Sciences, Hamadan, Iran; 3 Modeling of Noncommunicable Diseases Research Center, Department of Epidemiology & Biostatistics, School of Public Health, Hamadan University of Medical Sciences, Hamadan, Iran; Massachusetts General Hospital, United States of America

## Abstract

**Background:**

The effect of tranexamic acid (TXA) on bleeding and improvement of surgical field during functional endoscopic sinus surgery (FESS) is not clear yet. This study was conducted to answer this question.

**Methods:**

This trial was conducted on 60 patients with chronic sinusitis at Beasat Hospital, Hamadan, Iran, from April to November 2013. Thirty patients in the intervention group received three pledgets soaked with TXA 5% and phenylephrine 0.5% for 10 minutes in each nasal cavity before surgery. Thirty patients in the control group received phenylephrine 0.5% with the same way. The amount of bleeding and the quality of surgical field were evaluated at 15, 30, and 45 minutes after the start of surgery using Boezaart grading.

**Results:**

The quality of the surgical field in the intervention group compared to the control group was significantly better in the first quarter (P = 0.002) and the second quarter (P = 0.003) but not in the third quarter (P = 0.163). Furthermore, the amount of bleeding was much less during all periods in the intervention group than in the control group (P = 0.001).

**Conclusion:**

Topical TXA can efficiently reduce bleeding and improve the surgical field in FESS in patients with rhinosinusitis. Based on these findings, topical TXA may be a useful method for providing a suitable surgical field during the first 30 minutes after use.

**Trial Registration:**

Iranian Registry of Clinical Trials IRCT201212139014N15

## Introduction

Bleeding during endoscopic sinus surgery is still a challenge for surgeons and anesthesiologists [1]. Although extensive blood loss is rare during endoscopic surgery, however, establishing a favorite surgical field is often difficult. The reason is that even slight bleeding may distort the view of the endoscope and increase the occurrence of complications, including blindness, diplopia, damage to the internal carotid artery, the longer duration of surgery, or even inconclusive surgery [2]–[4].

Many techniques have been proposed to improve the field of functional endoscopic sinus surgery (FESS). Bipolar diathermy, packing, local vasoconstrictors, and induced hypotension are the most commonly used techniques [3], [5], [6]. Diathermy can lead to local mucosal damage and delayed bleeding [3]. Using topical vasoconstrictions can lead to hemodynamic instability especially in patients with a history of hypertension or ischemic heart disease. Induction hypotension exposes the patients to more anesthetic drugs and hence a higher risk of potential side effects. However, neither of these methods guarantees a desirable surgical field with no bleeding. Therefore, investigators are working on more effective and safer methods to reduce bleeding and hence to improve the field of endoscopic sinus surgery [7].

Activation of fibrinolysis during and after surgery is a well-known phenomenon. Many mechanisms associated with coagulation disorders, such as surgical trauma, blood loss and consumption of coagulation factors and platelets, using crystalloid and colloid given during and after surgery, hypothermia, acidosis, foreign materials, and etc. [8], [9]. In recent studies, systemic infusion of anti-fibrinlytic drugs have been used to reduce bleeding in various forms of surgery such as major orthopedic surgery, adeno-tonsillectomy, and endoscopic sinus surgery [7], [10]–[12].

Tranexamic acid (TXA) is a synthetic antifibrinolytic agent that binds to the lysine binding sites of plasmin and plasminogen (13). Saturation of the binding sites causes separation of plasminogen from superficial fibrin and hence prevents fibrinolysis [13], [14]. Any surgical procedure can cause a considerable tissue damage and hence trigger the release of enzymes, such as ‘tissue plasminogen activator’ that converts plasminogen to plasmin and activates fibrinolysis process. TXA can prevent fibrinolysis activity by inhibiting the activity of this enzyme [15].

Systemic infusion of TXA associated with several potential side effects such as nausea, vomiting, diarrhea, allergic dermatitis, dizziness, hypotension, seizures, impaired vision, achromatopsia (impaired color vision), and particularly thromboembolic events [16]. Several studies have been conducted on topical TXA in different types of surgery but no systemic absorption or side effects have been reported [13], [17], [18].

To date, limited trials have examined the effect of TXA on reduction of bleeding in FESS. There is no consensus on the efficacy of TXA and its effective dose in reducing bleeding [7], [19]–[21]. This trial aimed to assess the effect of topical TXA on bleeding and improvement of surgical field during FESS in patients with chronic sinusitis with or without polyposis.

## Materials and Methods

The protocol for this trial and supporting CONSORT checklist are available as supporting information; see Checklist S1 and Protocol S1.

This triple blind randomized clinical trial was conducted at Beasat Hospital, affiliated with Hamadan University of Medical Sciences, in the west of Iran, from April to November 2013. All patients were enrolled voluntarily and gave a written informed consent. The ethic committee of the university approved the consent procedure and the whole trial (D-P-9-35-16).

The eligible patients with chronic sinusitis with or without polyposis who referred to Ear-Nose-Throat clinic of Beasat Hospital were enrolled if they had the following criteria: (a) being candidate for FESS based on AAO-HNS criteria [22]; (b) age of 18 to 60 years; (c) hemoglobin >10 mg/dl; (d) normal clotting time (CT), bleeding time (BT), international normalized ratio (INR), prothrombin time (PT), Partial thromboplastin time (PTT). The patients with the following criteria were excluded from the trial: (a) having diathesis to hemorrhage such as hemophilia; (b) thrombosis; (c) acute or chronic renal failure; (d) using heparin during 48 hours before surgery; (e) using aspirin during fourteen days before surgery; (f) allergy to TXA; (g) cirrhosis; (h) chronic diseases such as hypertension, diabetes, and heart failure; (i) pregnancy; (j) color blind; (k) having a cardiac stent; (l) having a nasal tumor.

The patients in the intervention group received three pledgets soaked with TXA 5% and phenylephrine 0.5% for 10 minutes in each nasal cavity before surgery. The patients in the control group received three pads soaked with only phenylephrine 0.5% for 10 minutes in each nasal cavity before surgery. We did not use other topical agents during the course of sinus surgery to improve hemostasis.

The primary outcomes of interest were: (a) the quality of the surgical field at 15, 30, and 45 minutes after the start of surgery using Boezaart grading [23] with 0–5 scores; and (b) bleeding at 15, 30, and 45 minutes after the start of surgery using blood accumulated in the suction chamber after reducing the amount of normal saline used for washing and measurement of nasopharyngeal pack weight and converting the blood weight into ml. The secondary outcomes of interest included the potential side effects of the TXA such as: (a) nausea; (b) vomiting; (c) and impaired color vision 24 hours after surgery and three days later.

Before surgery, the paranasal sinus (PNS) CT scan was done for all patients and scoring was done [24]. In addition, for patients with polyposis, endoscopic grading was done [25]. The patients with polyposis received oral corticosteroid with the same type and dose for 10 days before surgery to reduce the inflammation of the polyposis and hence prevent massive bleeding.

Given the depth of anesthesia and anesthetic drugs on bleeding, all patients were treated under general anesthesia with the same method. For this purpose, the patients were monitored by electrocardiography and pulse oximetry. Patients' blood pressure was measured every three minutes. After premedication with fentanyl 0.1 ml/kg and midazolam 0.05–0.1 ml/kg, the induction of general anesthesia was done with lidocaine 0.5 mg/kg and propofol 2 mg/kg and atracurium 0.5 g/kg and then intubation was done with appropriate endotracheal tube. After head up position at level of 30 degrees, the patient was ready for surgery. All patients placed and maintained at the same position throughout the surgery. The general anesthesia was continued with O_2_ and N_2_O and infusion of propofol 1.5–4.5 mg/kg/hr to maintain the mean arterial pressure between 70 to 80 mmHg. Maintenance dose of atracurium was repeated every 20 minutes [26]. During surgery, ringer lactate was administered according to the amount of blood loss and the patient's weight. The quality of surgical field based on Boezaart grading was categorized (Table 1).

**Table 1:** Boezaart grading for categorizing the quality of surgical field.

Grade	Description
0	No bleeding (cadaveric conditions)
1	Slight bleeding: no suctioning required
2	Slight bleeding: occasional suctioning required
3	Slight bleeding: frequent suctioning required, bleeding threatens surgical field a few seconds after suction is removed
4	Moderate bleeding: frequent suctioning required and bleeding threatens surgical field directly after suction is removed
5	Severe bleeding: constant suctioning required; bleeding appears faster than can be removed by suction; surgical field severely threatened and surgery usually not possible

Alimian et al conducted a clinical trial and examined the effect of intravenous TXA on blood loss and the quality of the surgical field during endoscopic sinus surgery [7]. According the results of this trial, the bleeding score in the intervention and control groups was 14.3% and 42.9% respectively. Based on these results, we reached a sample of 30 for each group and a total sample of 60 at 95% significant levels and 80% statistical power.

The eligible patients were randomly assigned to the intervention and control groups using the balance block randomization method. For this purpose, we prepared six sheets of paper, writing on three sheets 'I' for 'Intervention' and on three ‘C’ for ‘control’. The paper sheets were pooled, placed in a container, randomly drawn out one at a time for each patient without replacement until all six sheets were drawn. The six paper sheets were then placed back into the container and this action repeated until the sample size of 60 was reached. The allocation remained concealed during the study.

The random allocation was conducted by a resident of surgery, who was the coordinator of the trial group. Thus, the surgeon, who evaluated the effect of interventions, were not aware of the administered drugs. The statistical analyst was unaware of the trial groups either, until the data were analyzed and the labels were decoded. The patients were unconscious during the surgery and thus, they knew nothing about the type of intervention they received. Accordingly the trial was run as a triple blind design.

The *t*-test was used for analysis of continuous variables and the chi-square test and Fisher exact test for nominal variables. All statistical analyses were performed at a significance level of 0.05 using Stata software version 11 (StataCorp, College Station, TX, USA).

## Results

**Figure 1:**
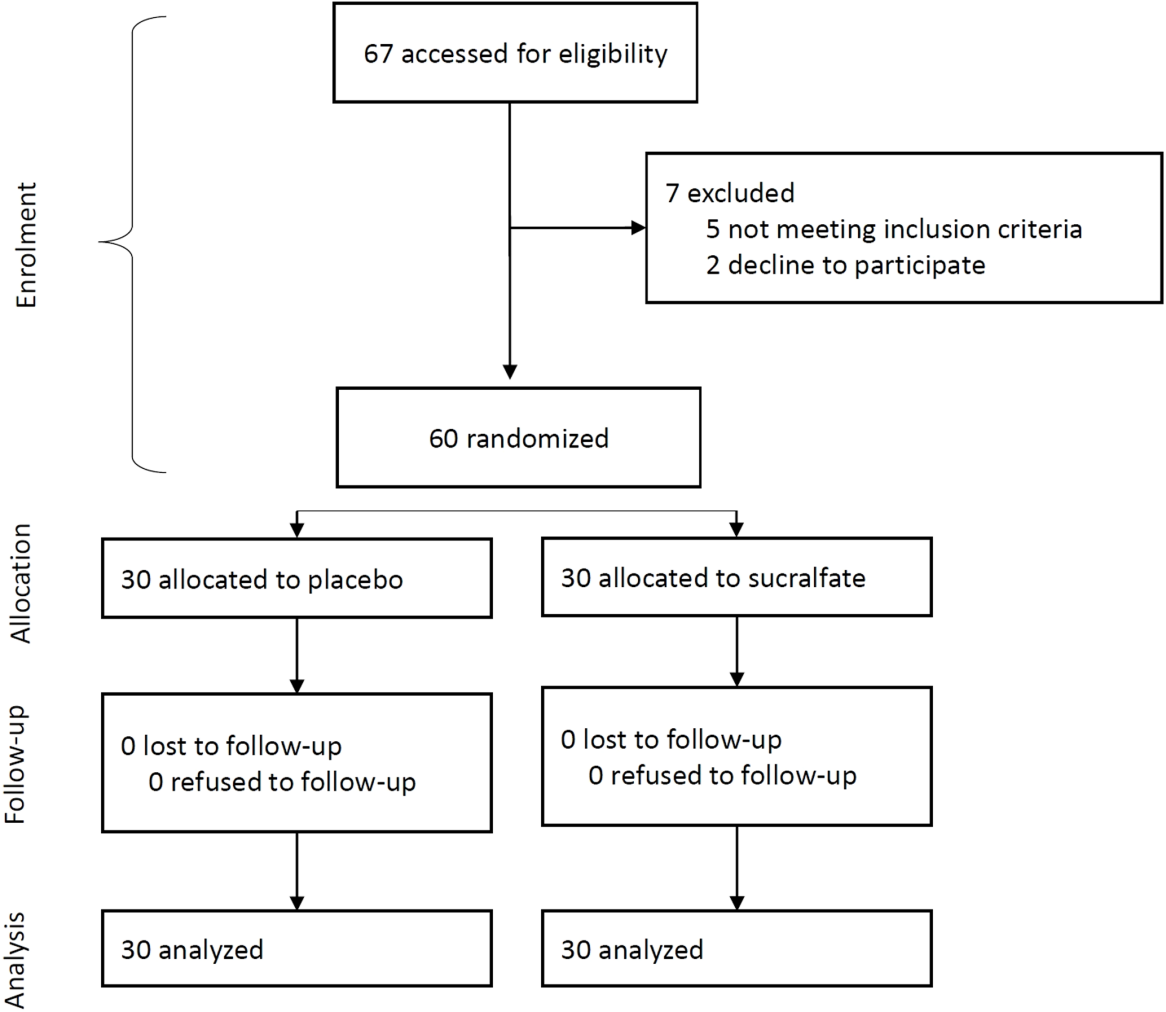
Flow diagram of the progress through the phases of the randomized trial of the two groups.

Of 67 patients identified, 5 were ineligible, 2 declined to participate. The randomization was based on the remaining 60 patients, of whom 30 patients were randomly allocated to the intervention group (TXA plus phenylephrine) and 30 patients to the control group (phenylephrine alone). No patient was lost to follow-up thus the analysis was based on data from 60 patients (Figure 1).

Thirty-six patients were males and 24 were females. The mean (SD) age of the patients was 35.77 (10.78) with a minimum and a maximum of 18 and 60 years respectively. The demographic and clinical characteristics of the intervention and control groups were similar with no statistically significant difference (Table 2).

**Table 2:** Distribution of the characteristics of the study population by groups, intervention (tranexamic acid plus phenylephrine) versus control (phenylephrine alone).

	Intervention (n = 30)		Control (n = 30)		
Characteristics	Number	Percent	Number	Percent	*P* value
Gender					0.292
Male	16	53.3	20	66.7	
Female	14	46.7	10	33.3	
Previous functional endoscopic sinus surgery					0.278
Yes	6	20.0	3	10.0	
No	24	80.0	27	90.0	
Polyposis					0.580
None	9	30.0	12	40.0	
Grade I	9	30.0	11	36.7	
Grade II	10	33.3	6	20.0	
Grade III	2	6.7	1	3.3	
**Characteristics**	**Mean**	**SD**	**Mean**	**SD**	***P*** ** value**
Age (yr)	37.43	11.75	34.10	9.61	0.234
Duration of surgery (min)	114.00	37.93	102.73	25.36	0.182
Systolic blood pressure (mmHg)	100.27	7.48	102.83	7.90	0.201
Diastolic blood pressure (mmHg)	62.93	9.76	67.60	9.22	0.062
Heart rate (/min)	79.33	8.53	76.23	8.95	0.175
Mean arterial pressure (mmHg)	75.27	8.17	79.27	8.71	0.072
Total score (CT scan)	16.90	5.74	16.93	5.32	0.982
Hemoglobin (mg/dl)	14.33	1.65	14.36	1.52	0.955
Hematocrit (%)	44.13	0.70	43.69	4.17	0.667
Bleeding time (min)	2.00	0.49	1.84	0.55	0.242
Prothrombin time (s)	12.37	0.49	12.43	0.57	0.628
Partial thromboplastin time (s)	32.60	2.74	33.53	2.67	0.187
International normalization ratio	1.04	0.05	1.04	0.05	1.000
Platelet count (/mcl)	287166.70	95965.54	275633.30	67941.90	0.593

The quality of surgical field based on Boezaart grading in the two groups at 15, 30 and 45 minutes after the start of surgery is given in Table 3. At 15 minutes after the start of surgery, the majority (76.7%) of the patients in the intervention group were in grade II whereas, at the same time, only 43.3% of the patients in the control group were in grade II. Furthermore, no patient in the intervention group was in grade IV while 13.3% of the patients in the control group were in grade IV (*P* = 0.002).

**Table 3:** The effect of tranexamic acid plus phenylephrine versus phenylephrine alone on the quality of surgery field by duration of time after surgery based on Boezaart grading.

	Intervention (n = 30)		Control (n = 30)		
Quality of surgery field (grade)^a^	Number	Percent	Number	Percent	*P* value
**0–15 min**					0.002
Grade I	3	10.0	0	0.0	
Grade II	23	76.7	13	43.3	
Grade III	4	13.3	13	43.3	
Grade IV	0	0.0	4	13.3	
**16–30 min**					0.003
Grade I	1	3.3	0	0.0	
Grade II	21	70.0	8	26.7	
Grade III	7	23.3	16	53.3	
Grade IV	1	3.3	6	20.0	
**31–45 min**					0.163
Grade I	1	3.5	0	0.0	
Grade II	14	48.3	7	24.1	
Grade III	11	37.9	17	58.6	
Grade IV	3	10.3	5	17.2	

a Grades of 0 and V were not seen.

At 30 minutes after the start of surgery, the majority (70.0%) of the patients in the intervention group and only 26.7% of the patients in the control group were in grade II. The majority (53.3%) of the control group were in grade III (*P* = 0.003). At 45 minutes after the start of surgery, most (48.3%) of the patients in the intervention group were in grade II while most of the patients in the control group were in grade III, but the difference was not statically significant (*P* = 0.163).

The amount of bleeding in the intervention and control groups at 15, 30 and 45 minutes after the start of surgery is given in Table 4. The amount of bleeding during all periods was much higher in the control group than in the intervention group. The overall (whole time) amount of bleeding was on average 100.10 ml in the intervention group and 170.49 ml in the control group (*P* = 0.001).

**Table 4:** The effect of tranexamic acid plus phenylephrine versus phenylephrine alone on the amount of bleeding by duration of time after surgery using ANOVA test.

	Intervention (n = 30)		Control 2 (n = 30)		
Amount of bleeding (ml)	Mean	SD	Mean	SD	*P* value^a^
0–15 min	23.37	13.92	48.67	14.53	0.001
16–30 min	34.50	21.49	60.10	18.78	0.001
31–45 min	42.38	23.77	60.38	23.17	0.006
Total time	100.10	52.50	170.49	45.87	0.001

a Analysis of variance adjusted for polyposis and treatment-by-polyposis interaction (the interaction term was significant for no group).

Thirty-nine out of 60 patients had polyposis of different grades, 21 in the intervention group and 18 in the control group. The quality of the surgical field and amount of bleeding in the intervention and control groups was evaluated in patients with and without polyposis separately (Table 5 and 6). According to these results, in patients with or without polyposis, the quality of surgical field was much better in the intervention group than in the control group during all periods. However, there was no statistically significant difference between the two groups in patients with polyposis.

**Table 5:** Effect of tranexamic acid plus phenylephrine versus phenylephrine alone on quality of surgery field by duration of time and with or without polyposis using Fisher exact test.

Quality of surgery field (grade)^a^	Without polyposis (n = 21)			With polyposis (n = 39)		
	Intervention	Control	*P* value	Intervention	Control	*P* value
**0–15 min**			0.010			0.085
Grade I	0	0		3	0	
Grade II	8	3		15	10	
Grade III	1	7		3	6	
Grade IV	0	2		0	2	
**16–30 min**			0.003			0.111
Grade I	0	0		1	0	
Grade II	8	2		13	6	
Grade III	1	8		6	8	
Grade IV	0	2		1	4	
**31–45 min**			0.014			0.951
Grade I	0	0		1	0	
Grade II	7	2		7	5	
Grade III	2	9		9	8	
Grade IV	0	1		3	4	

a Grades of 0 and V were not seen.

**Table 6:** Effect of tranexamic acid plus phenylephrine versus phenylephrine alone on amount of bleeding by duration of time and with or without polyposis using Fisher exact test.

Amount of bleeding (mL) mean (SD)	Without polyposis (n = 21)			With polyposis (n = 39)		
	Intervention	Control	*P* value	Intervention	Control	*P* value
0–15 min	21.11 (6.49)	52.58 (11.12)	0.001	24.33 (16.15)	46.06 (16.19)	0.001
16–30 min	27.67 (16.44)	63.42 (20.60)	0.001	37.43 (23.05)	57.89 (17.73)	0.004
31–45 min	32.78 (16.19)	60.08 (17.91)	0.002	46.70 (25.67)	60.59 (26.82)	0.117
Total	81.56 (32.38)	176.08 (38.52)	0.001	108.45 (58.17)	166.53 (51.21)	0.003

The potential side effects of TXA such as nausea, vomiting, and impaired color vision were evaluated 24 hours after surgery and three days later. But no side effect was reported.

## Discussion

FESS is usually done for the treatment of patients with chronic sinonasal disease who do not respond to the conventional medical treatment. Good visibility during FESS is necessary because nasal tiny anatomical structures, which are full of vessels, limit the nasal endoscopic access. In such situation, even a minor bleeding can lead the surgical procedure left unfinished [27].

Systemic infusion of antifibrinolytic drugs effectively reduces bleeding within and after surgery [7], [11], [12]. However, systemic infusion of fibrinolysis inhibitors can increase the tendency to thrombosis and thus the risk of thromboembolism. To avoid or at least reduce the risk of thromboembolism, topical TXA has been used in various surgical procedures [28]. TXA or trans-4-aminomethylcyclohexane carboxylic acid is a valuable antifibrinolytic agent that has been used for many years [29]. To date, many efforts have been made in the use of TXA to reduce bleeding and to improve the surgical field in FESS [7], [19]–[21]. TXA is normally used intravenously. However, it is extensively used topically by several researchers in various kinds of surgical procedures. Nonetheless, yet there is no consensus on its effective dose [13], [17], [18], [20], [30].

The evidence showed that oral and intravenous TXA can reduce blood loss and improve surgical field [7], [21]. To the best of our knowledge, two trials have been conducted to investigate the effect of topical TXA on bleeding and improvement of surgical field in FESS. Athanasiadis et al [19] designed a randomized controlled trial to examine the effect of topical epsilon-aminocaproic acid (EACA) and TXA on bleeding in the surgical field during FESS. In this study, 30 patients were randomized to receive either 2.5 g of EACA, 100 mg of TXA, or 1 g of TXA whereas the contralateral side received saline. They concluded that topical application of TXA could efficiently reduce bleeding and improve the surgical field. The results of this trial were consistent with our results.

Jabalameli et al [20] conducted a clinical trial on 56 patients to assess the effects of topical TXA on improving surgical field and hemostasis. In this trial, 26 patients received topical TXA and 30 patients received placebo. According to the results of this study, the amount of bleeding in the TXA group was less than the placebo group (174.0±10.6 vs 229.1±23.8 ml; P<0.05). Furthermore, the frequency of score 3 was 26% in TXA group and 70% in the placebo group (70%). They concluded that topical application of TXA can reduce intraoperative bleeding in FESS. However, the way of drug prescription was not explained clearly. The effect of TXA was not examined in different periods of time after surgery either. Whereas in our study, we assessed the effect of TXA on bleeding and surgical field in different periods of time (15, 30, and 45 minutes) and indicated that the TXA had a significant effect on blood loss and improvement of surgical field at 15 and 30 minutes after the start of surgery but a non-significant effect thereafter. This may be attributed to the reduction in local concentration of TXA. Furthermore, we examined the effect of TXA on blood loss and quality of surgical field in patients with and without polyposis separately. We showed that TXA could provide an effective hemostasis and a suitable surgical field in patients without polyposis compared to control group. However, TXA had limited effect on bleeding and quality of surgical field in patients with polyposis. This may be either attributed to the hemorrhagic nature of polyposis which may overcome the hemostatic effect of TXA or it may be that the study was underpowered to show the difference in subgroups because the study did not have enough polyps patient to reach statistical significance. Furthermore, the total number of the patients with polyposis was 39; 21 of which were in the intervention group and 18 in the control group. That means the number of polyp patients and hence the risk of hemorrhage was higher in the intervention group than the control group. If the proportion of the polyposis was the same in the two groups, the difference between the two groups might become much greater and hence the statistical power of our study might become stronger. This issue favor our findings that topical TXA can efficiently reduce bleeding and improve the surgical field in FESS in patients with rhinosinusitis

The main limitation of this study was the small sample size. This might introduce random error in the results of subgroup analysis of patients with and without polyposis. Therefore, we suggest that randomized controlled trials are designed to examine the effect of TXA on bleeding and quality of surgical field in patients with and without polyposis separately. This would be an important area for a future adequately powered study for patients with polyps.

## Conclusion

Topical TXA can efficiently reduce bleeding and improve the surgical field in FESS in patients with rhinosinusitis. Based on these findings, topical TXA may be a useful method for providing a suitable surgical field during the first 30 minutes after use. However, additional trials should be conducted to explore TXA efficacy in different subgroups of the patients with or without polyposis.

## Supporting Information

Protocol S1 Trial Protocol.
(DOCX)

Checklist S1 CONSORT Checklist.
(PDF)
