# Supplementary material for: Effect of Topical Tranexamic Acid on Bleeding and Quality of Surgical Field during Functional Endoscopic Sinus Surgery in Patients with Chronic Rhinosinusitis: A Triple Blind Randomized Clinical Trial
Source: PLoS One. 2014 Aug 18;9(8):e104477. doi: 10.1371/journal.pone.0104477 (PMC4136784; doi:10.1371/journal.pone.0104477)
Supplement: Protocol S1 — Trial Protocol. (DOCX) [file pone.0104477.s001.docx]

**Dear Editor-in-Chief,**

Subject: Supplement A

This supplement contains the following items:

1. Original protocol and statistical analysis plan
2. Final protocol and statistical analysis plan
3. Summary of changes

Sincerely yours,

**Jalal Poorolajal (MD, PhD)**

Associate Professor of Epidemiology, Modeling of Noncommunicable Diseases Research Center, Department of Epidemiology & Biostatistics, School of Public Health, Hamadan University of Medical Sciences, Hamadan, Iran

E-mail 1: poorolajal@umsha.ac.ir

E-mail 2: poorolajal@yahoo.com

**Original protocol and statistical analysis plan**

Effect of local tranexamic acid versus phenylephrine on reduction of bleeding and improvement of surgical filed during functional endoscopic sinus surgery in patients with chronic rhinosinusitis: a double blinded randomized controlled trial

**Objectives**: To assess the effect of local tranexamic acid on reduction of bleeding and improvement of surgical filed during functional endoscopic sinus surgery in patients with chronic rhinosinusitis.

**Design**: Double blinded randomized controlled trial.

**Setting and conduct**: The eligible patients with chronic rhinosinusitis who will refer to ENT clinic of Besat hospital in 2013.

**Inclusion criteria**: (a) patients with chronic rhinosinusitis with or without polyposis; (b) age of 18 to 60 years; (c) hemoglobin >10 mg/dl; (d) normal CT, BT, INR, PT, PTT.

**Exclusion criteria**: (a) having hemorrhagic abnormality such as hemophilia; (b) thrombosis; (c) acute or chronic renal failure; (d) using heparin during 48 hours before surgery; (e) using aspirin during three days before surgery; (f) allergy to tranexamic acid; (g) cirrhosis; (h) chronic diseases such as hypertension, diabetes, and heart failure; (i) pregnancy; (j) color blind; (k) having cardiac stent; (l) having nasal tumor.

**Intervention**: 30 patients will receive three pads impregnated with tranexamic acid 5% and phenylephrine 0.5% for 10 minutes in each nasal cavity before surgery.

**Control**: 30 patients will receive three pads impregnated only with phenylephrine 0.5% for 10 minutes in each nasal cavity before surgery

**Primary outcomes**: (a) quality of surgical field at 15, 30, and 45 minutes after surgery using Boezaart grading with1-5 scores; (b) bleeding at 15, 30, and 45 minutes after surgery using blood accumulated in the suction chamber after reducing the amount of serum used for washing and measurement of nasopharyngeal pack weight and converting the blood weight into ml.

**Secondary outcomes**: measurement of (a) nausea; (b) vomiting; (c) and impaired color vision 24 hours after surgery and three days later.

**Gender**: Both male and female

**Age**: 18-60 years

**Condition ICD-10 code**: J32 Chronic sinusitis

**Ethical approval**: Research Ethic Committee of Hamadan University of Medical Sciences

**Date**: 2013-04-09

**Number**: D/P/9/35/16

**IRCT registration number:** IRCT201212139014N15

**Expected recruitment start date**: 2013-06-22

**Expected recruitment end date**: 2013-12-22

**Statistical analysis plan**

The t-test was used for analysis of continuous variables and chi-square test and Fisher exact test for nominal variables. All statistical analyses were performed at 0.05 significance level using statistical software Stata 11 (StataCorp, College Station, TX, USA).

**Final protocol and statistical analysis plan**

Effect of local tranexamic acid plus phenylephrine versus phenylephrine alone on reduction of bleeding and improvement of surgical filed during functional endoscopic sinus surgery in patients with chronic rhinosinusitis: a double blinded randomized controlled trial

**Objectives**: To assess the effect of local tranexamic acid on reduction of bleeding and improvement of surgical filed during functional endoscopic sinus surgery in patients with chronic rhinosinusitis.

**Design**: Double blinded randomized controlled trial.

**Setting and conduct**: The eligible patients with chronic rhinosinusitis who will refer to ENT clinic of Besat hospital in 2013.

**Inclusion criteria**: (a) patients with chronic rhinosinusitis with or without polyposis; (b) age of 18 to 60 years; (c) hemoglobin >10 mg/dl; (d) normal CT, BT, INR, PT, PTT.

**Exclusion criteria**: (a) having hemorrhagic abnormality such as hemophilia; (b) thrombosis; (c) acute or chronic renal failure; (d) using heparin during 48 hours before surgery; (e) using aspirin during three days before surgery; (f) allergy to tranexamic acid; (g) cirrhosis; (h) chronic diseases such as hypertension, diabetes, and heart failure; (i) pregnancy; (j) color blind; (k) having cardiac stent; (l) having nasal tumor.

**Intervention**: 30 patients will receive three pads impregnated with tranexamic acid 5% and phenylephrine 0.5% for 10 minutes in each nasal cavity before surgery.

**Control**: 30 patients will receive three pads impregnated only with phenylephrine 0.5% for 10 minutes in each nasal cavity before surgery

**Primary outcomes**: (a) quality of surgical field at 15, 30, and 45 minutes after surgery using Boezaart grading with1-5 scores; (b) bleeding at 15, 30, and 45 minutes after surgery using blood accumulated in the suction chamber after reducing the amount of serum used for washing and measurement of nasopharyngeal pack weight and converting the blood weight into ml.

**Secondary outcomes**: measurement of (a) nausea; (b) vomiting; (c) and impaired color vision 24 hours after surgery and three days later.

**Gender**: Both male and female

**Age**: 18-60 years

**Condition ICD-10 code**: J32 Chronic sinusitis

**Ethical approval**: Research Ethic Committee of Hamadan University of Medical Sciences

**Date**: 2013-04-09

**Number**: D/P/9/35/16

**IRCT registration number:** IRCT201212139014N15

**Expected recruitment start date**: 2013-06-22

**Expected recruitment end date**: 2013-12-22

**Statistical analysis plan**

The t-test was used for analysis of continuous variables and chi-square test and Fisher exact test for nominal variables. All statistical analyses were performed at 0.05 significance level using statistical software Stata 11 (StataCorp, College Station, TX, USA).

**Summary of changes**

Effect of local tranexamic acid plus phenylephrine versus phenylephrine alone on reduction of bleeding and improvement of surgical filed during functional endoscopic sinus surgery in patients with chronic rhinosinusitis: a triple blinded randomized controlled trial
